# Three challenges that the COVID-19 pandemic represents for psychiatry

**DOI:** 10.1192/bjp.2020.106

**Published:** 2020-05-15

**Authors:** Ladislav Kesner, Jiří Horáček

**Affiliations:** 1 Department of Applied Neurosciences and Neuroimaging, National Institute of Mental Health, Klecany, Czech Republic; 2 National Institute of Mental Health, Klecany, Czech Republic

**Keywords:** COVID-19, uncertainty, negative mood, media, mental health

## Abstract

The evolving COVID-19 pandemic and its likely consequences add to the already substantial psychosocial burden caused by global problems, existential threats and heightened uncertainty, which are increasingly confronting communities worldwide. Here we briefly outline three challenges for clinical psychiatry and research, related to coping with the social epidemiology of negative moods, stress and socially mediated traumatic experiences brought on by these adverse developments.

As the COVID-19 pandemic spreads around the globe, it is fast becoming a major emergency for psychiatry and the entire field of mental health. Enormous stress, negative emotions and a sense of uncertainty are adversely affecting people with pre-existing mental health conditions by causing exacerbation of symptoms, triggering relapses and acting as a potential trigger for new-onset illness. The mental health consequences might be particularly serious not just for patients with severe COVID-19 infection, but also for their carers and the health professionals who must bear the brunt of the crisis.^1^ Immediate and specifically targeted mental health interventions must therefore be part of the overall public health response to the crisis.^2^ As the pandemic unfolds, with as yet unforeseeable consequences, it seems increasingly likely that, in addition to medical, economic and social implications, it will exert a long-term impact on mental health globally. Even when the outbreak is ultimately contained, the psychosocial repercussions of enforced social distancing, the disruption of normal behavioural patterns, and major changes to the established norms of individual and collective behaviour, aggravated by economic disruptions and the gradual adjustment to the ‘new normal’, will likely affect the mental well-being of global communities on a scale unprecedented in recent history.

In addition to the direct impact of this public health emergency on mental health services, the crisis also presents a challenge for updating the current conceptual framework of psychiatry. We argue that the lesson of the COVID-19 pandemic can inspire mental health services, and psychiatry research, in three principal domains.

## COVID-19 and other global adverse developments

First, it must be emphasised that, even though it is an unprecedented health threat, the COVID-19 outbreak is not a stand-alone phenomenon. It arrived at a time when global communities and societies have, over the past decade, already been increasingly saturated with negative emotions, fears, anxieties and feelings of helplessness in response to a perception of serious global problems – from deepening inequality, the expected disastrous consequences of climate change, the proliferation of different forms of extremism, such as terrorism, the unexpected rise of authoritarian political tendencies and regimes, the migration crisis and the accompanying steep rise in xenophobia and polarisation of societies. It is likely that the converging negative nature of these problems will substantially affect mental health not in a purely additive sense, but with a more serious multiplicative effect. Therefore, psychiatry as a discipline should start to prepare effective strategies to deal with the entire cluster of these interlinked problems.

While commentators and social scientists have paid attention to these developments, psychiatry has not yet sufficiently acknowledged how mental health is affected by the pressure caused by rapidly escalating problems and the threats confronting global communities, or how individual mental health is dynamically constrained and affected by interactions between individual minds and brains in a social space. This is an inherently difficult perspective for the model of biomedical psychiatry today to embrace, embedded as it is in an individual-level perspective on brain disease. This model will increasingly prove incapable of dealing with the challenges ushered in by adverse global developments.

## The mediating role of public representations

Second, while ultimately it is true that mental health professionals will always need to provide care to individuals with their specific needs, it is imperative to recognise that in a world perceived as volatile, unpredictable and filled with existential threats, a growing number of individual cases of anxiety, mood and other mental disorders are embedded and causally linked to the socially mediated and transmitted negative feelings and emotions connected to coping with such threats. The psychobiological aetiological factors for anxiety and mood disorders should be newly understood as also arising at this social environment level, rather than at the genetic, molecular or neural circuitry level. At the same time, it must be recognised that adverse social and traumatic experiences increasingly do not just stem from individual life experiences but are sustained by publicly transmitted and shared negative mental content. The unprecedented increase in connectivity between people and societies by means of the internet, electronic communication and, first and foremost, social media may play a principal role in forcing the spread of negatively valenced news and in deepening the impact that objectively existing problems have on mental health as a consequence. Therefore, of particular importance at present is to obtain a better understanding of the processes through which social stressors and traumatic experiences are transmitted by public representations – most notably by media news – and then shared in the social space.^3^ Hence, the mediatory role of the media on mental health should be intensively studied as an indispensable prerequisite for developing effective prevention strategies to mitigate negative impacts.

## Intolerance of uncertainty

Third, the common denominator of all the above-mentioned social burdens, of which the COVID-19 pandemic is the latest and most dramatic, is the heightened level of uncertainty they create. Intolerance of uncertainty has been highlighted as a basic transdiagnostic mechanism that underlies the development of different anxiety, mood and psychotic disorders as well as maladaptive coping styles, psychological defences such as avoidance behaviours, perceived burdensomeness, aggression and suicidality.^4^ On the other hand, the fact that anxiety and stress may facilitate and accelerate the development of new, adaptive coping styles in response to negative events^5^ opens up possibilities for effective interventions on both the individual and the society-wide levels.

## Conclusion

The COVID-19 pandemic is a wake-up call to psychiatry as a clinical and scientific discipline, summoning it to team up with the social sciences to address the social epidemiology of negative moods, stress, anxiety and helplessness, even as psychiatrists themselves have little means of directly influencing the social parameters of its transmission. To do so psychiatry urgently needs to embrace the notion that (disordered) mental states have their collective dimension and indeed it should seek to understand how the individual and the collective are mutually constrained.

## Supplementary material

For supplementary material accompanying this paper visit http://dx.doi.org/10.1192/bjp.2020.106. click here to view supplementary material
